# Hybridizing five neural-metaheuristic paradigms to predict the pillar stress in bord and pillar method

**DOI:** 10.3389/fpubh.2023.1119580

**Published:** 2023-01-24

**Authors:** Jian Zhou, Yuxin Chen, Hui Chen, Manoj Khandelwal, Masoud Monjezi, Kang Peng

**Affiliations:** ^1^School of Resources and Safety Engineering, Central South University, Changsha, China; ^2^School of Geological and Mining Engineering, Xinjiang University, Urumqi, China; ^3^Institute of Innovation, Science and Sustainability, Federation University Australia, Ballarat, VIC, Australia; ^4^Faculty of Engineering, Tarbiat Modares University, Tehran, Iran

**Keywords:** pillar stress, BP neural network, meta-heuristic algorithms, prediction model, optimization

## Abstract

Pillar stability is an important condition for safe work in room-and-pillar mines. The instability of pillars will lead to large-scale collapse hazards, and the accurate estimation of induced stresses at different positions in the pillar is helpful for pillar design and guaranteeing pillar stability. There are many modeling methods to design pillars and evaluate their stability, including empirical and numerical method. However, empirical methods are difficult to be applied to places other than the original environmental characteristics, and numerical methods often simplify the boundary conditions and material properties, which cannot guarantee the stability of the design. Currently, machine learning (ML) algorithms have been successfully applied to pillar stability assessment with higher accuracy. Thus, the study adopted a back-propagation neural network (BPNN) and five elements including the sparrow search algorithm (SSA), gray wolf optimizer (GWO), butterfly optimization algorithm (BOA), tunicate swarm algorithm (TSA), and multi-verse optimizer (MVO). Combining metaheuristic algorithms, five hybrid models were developed to predict the induced stress within the pillar. The weight and threshold of the BPNN model are optimized by metaheuristic algorithms, in which the mean absolute error (MAE) is utilized as the fitness function. A database containing 149 data samples was established, where the input variables were the angle of goafline (A), depth of the working coal seam (H), specific gravity (G), distance of the point from the center of the pillar (C), and distance of the point from goafline (D), and the output variable was the induced stress. Furthermore, the predictive performance of the proposed model is evaluated by five metrics, namely coefficient of determination (R^2^), root mean squared error (RMSE), variance accounted for (VAF), mean absolute error (MAE), and mean absolute percentage error (MAPE). The results showed that the five hybrid models developed have good prediction performance, especially the GWO-BPNN model performed the best (Training set: R^2^ = 0.9991, RMSE = 0.1535, VAF = 99.91, MAE = 0.0884, MAPE = 0.6107; Test set: R^2^ = 0.9983, RMSE = 0.1783, VAF = 99.83, MAE = 0.1230, MAPE = 0.9253).

## 1. Introduction

The bord and pillar method has the advantages of less equipment investment, flexible equipment operation, fast production, short construction period, and simple support. It is widely used in the United States, Australia, India, South Africa, and other countries, and is the dominant method of coal mining. Among them, the stability of the pillar determines the stability of the room-and-pillar goaf site and is one of the prerequisites for the safe working conditions of the room-and-pillar mine (1). Pillar design is the key to the success of bord and pillar coal mining. Estimating the stress at different positions in a pillar is very important to work out the pillar size under the coal pillar strength conditions (2). The mining induced stresses of the pillars constantly change and are highly influenced by the dynamics of the formation equilibrium during the development and depilating phases of coal mining.

There are various methods to evaluate mining-induced stresses on coal pillars, such as empirical (3–7) and numerical methods (8–12). An empirical method is a method of inductive analysis by collecting some previous cases. Singh et al. (13) modified the earlier developed empirical relationship to estimate the extent of influence and the value of the final mining-induced stress on a coal pillar. Although these empirical formulas are used to predict pillar stress, they take fewer factors into account and have been validated in only a few engineering sites, and cannot be applied well beyond the original environmental characteristics. Numerical modeling techniques based on the finite element method (FEM), boundary element method (BEM), finite difference method (FDM), and discrete element method, etc. are more advantageous than the empirical methods in the complex stress conditions caused by coal seam mining with compound geometric shapes (14). Some researchers used numerical simulation methods to study pillar stress state, pillar stability, and other characteristics (15–21). However, despite the low cost and ease of operation of numerical methods, there are many assumptions in the simulation process that require simplified boundary conditions and material properties, leading to idealized results that differ from the actual ones. In addition, the results and accuracy vary due to the different forms of structural discretization, which does not guarantee the accuracy of predictions, and it is difficult to successfully apply this specially developed model to other situations.

Recently, various machine learning algorithms have been increasingly used in the engineering field and have shown excellent predictive performance (21–32). A study by Cavaleri et al. (33) demonstrated the good performance of BPNN in predicting the average surface roughness of EDM surfaces. Psyllaki et al. (34) used artificial neural networks (ANN) to conduct the corresponding study. Armaghani et al. (35) constructed a hybrid model of a particle swarm optimization neural network to predict the settlement of pile foundations. Lu et al. (36) used tree prediction models and as well as feature selection techniques to predict the punching shear capacity of steel fiber reinforced concrete. Asteris et al. (37–40) also used different machine learning models for prediction in their study and the results proved that these machine learning models have excellent prediction performance. The values of induced stresses on coal pillars are related to several factors, and the influencing factors are complex non-linear relationships with each other. Machine learning can overcome the limitations caused by the non-linear high-dimensional problems involved in engineering, has powerful data processing capabilities, and develops machine learning algorithm models with high applicability and flexibility. Based on this, machine learning algorithms have been used to predict the relative performance of pillars. Zhou et al. (41) applied Support Vector Machines (SVMs) to determine pillar stability for underground mines selected from various coal and stone mines by using various indicators, such as width (W), height (H), W/H ratio, uniaxial compressive strength of rock and pillar stress. It was found that the SVMs showed good performance and can be applied as a practical tool for predicting pillar stability. Ahmad et al. (42) proposed random trees and C4.5 decision trees algorithms to predict pillar stability in underground coal and quarry mines, and both models were able to predict pillar stability with reasonable accuracy. Liang et al. (43) used Gradient Boosting Decision Tree (GBDT), Extreme Gradient Boosting (XGBoost), and Light Gradient Boosting Machine (LightGBM) algorithms to predict hard rock pillar stability and found good prediction capability of the models. Monjezi et al. (2) used an artificial neural network (ANN) to predict the stress of the mine pillar in the room-pillar method and found the coefficient of determination (R^2^) 0.988 between the calculated and predicted pillar stresses. The results were also compared with the BEM and found that the predictive competence of the artificial neural network was far better than that of the BEM numerical solution.

Currently, neural-metaheuristic hybrid models are considered and developed in many fields with high reliability (44–50). Therefore, in this study, five hybrid models were constructed to estimate the induced stresses at different locations within the coal pillar by combining back-propagation neural network (BPNN) and metaheuristic algorithms. Since the selection of weights and thresholds of BPNN has a great impact on the network training, and the random selection of hyper-parameters can lead to unstable prediction performance of BPNN models, the optimal weights and thresholds are selected by optimization techniques to ensure that the models have the best performance. The five metaheuristics selected for this study are the sparrow search algorithm (SSA), gray wolf optimizer (GWO), butterfly optimization algorithm (BOA), tunicate swarm algorithm (TSA), and multi-verse optimizer (MVO).

## 2. Methods

### 2.1. Sparrow search algorithm

SSA was inspired by the foraging behavior and anti-predation behavior of sparrows (51) and has been applied to solve many complex engineering optimization problems (52–55). A brief description of SSA is as follows.

Sparrow population members are divided into three categories: finders, entrants, and scouts. The role of finders is to find food, providing all entrants with a foraging area and direction. The position update of the finder during each iteration is described by Equation (1).

(1) $$\begin{array}{l} X_{ij}^{t+1}=\{\begin{array}{ll} X_{ij}^{t}\cdot \exp\left(\frac{-i}{\alpha \cdot T_{max}}\right), & R_{2}<ST\\ X_{ij}^{t}+Q\cdot L, & R_{2}\geq ST \end{array} \end{array}$$

where *t* represents the current iteration number, *T*_max_ is the maximum number of iterations, $X_{ij}^{t}$ is the position of the *i* sparrow in the *j* dimension, α is a uniform random number between [0, 1], *Q* is a random number that follows a standard normal distribution, *L* is a 1 × *d* matrix whose elements are all 1. *R*_2_ ∈ [0, 1], represents the warning value; *ST* ∈ [0.5, 1], represents the safety value.

Entrants will follow the finders for foraging in order to obtain food, and its position update is described by Equation (2).

(2) $$\begin{array}{l} X_{ij}^{t+1}=\{\begin{array}{l} Q\cdot \exp\left(\frac{X_{w}^{t}-X_{ij}^{t}}{i^{2}}\right),\;i>\frac{N}{2}\\ X_{p}^{t+1}+|X_{ji}^{t}-X_{p}^{t+1}|A^{+}L,\;\text{otherwise} \end{array} \end{array}$$

where $X_{p}^{t+1}$ represents the best position of the finder at the *t* + 1 iteration; $X_{w}^{t}$ represents the global worst position at the t iteration; *A* is a matrix of size 1 × *d* with elements randomly assigned to 1 or −1, and *A*^+^ = *A*^*T*^(*AA*^*T*^)^−1^; *N* is the population size.

Scouts are responsible for reconnaissance and early warning, alerting the entire population to give up foraging when danger is detected. The position update of the scout is described by Equation (3).

(3) $$\begin{array}{l} X_{ij}^{t+1}=\{\begin{array}{ll} X_{b}^{t}+\beta \cdot |X_{ij}^{t}-X_{b}^{t}|, & f_{i}>f_{g}\\ X_{ij}^{t}+K\cdot \left(\frac{\left|X_{ij}^{t}-X_{w}^{t}\right|}{\left(f_{i}-f_{w}\right)+\varepsilon }\right), & f_{i}=f_{g} \end{array} \end{array}$$

Where $X_{b}^{t}$ represents the global optimal position at the t iteration; β is the step size parameter, which obeys the normal distribution random number with mean 0 and variance 1; *K* is a random number in the range [−1, 1]. *f*_*i*_, *f*_*w*_ and *f*_*g*_ are the individual fitness value, the global worst fitness value, and the global optimal fitness value of the current sparrow, respectively. ε is an extremely small constant that prevents the denominator from being zero.

### 2.2. Gray wolf optimizer

**Figure 1:**
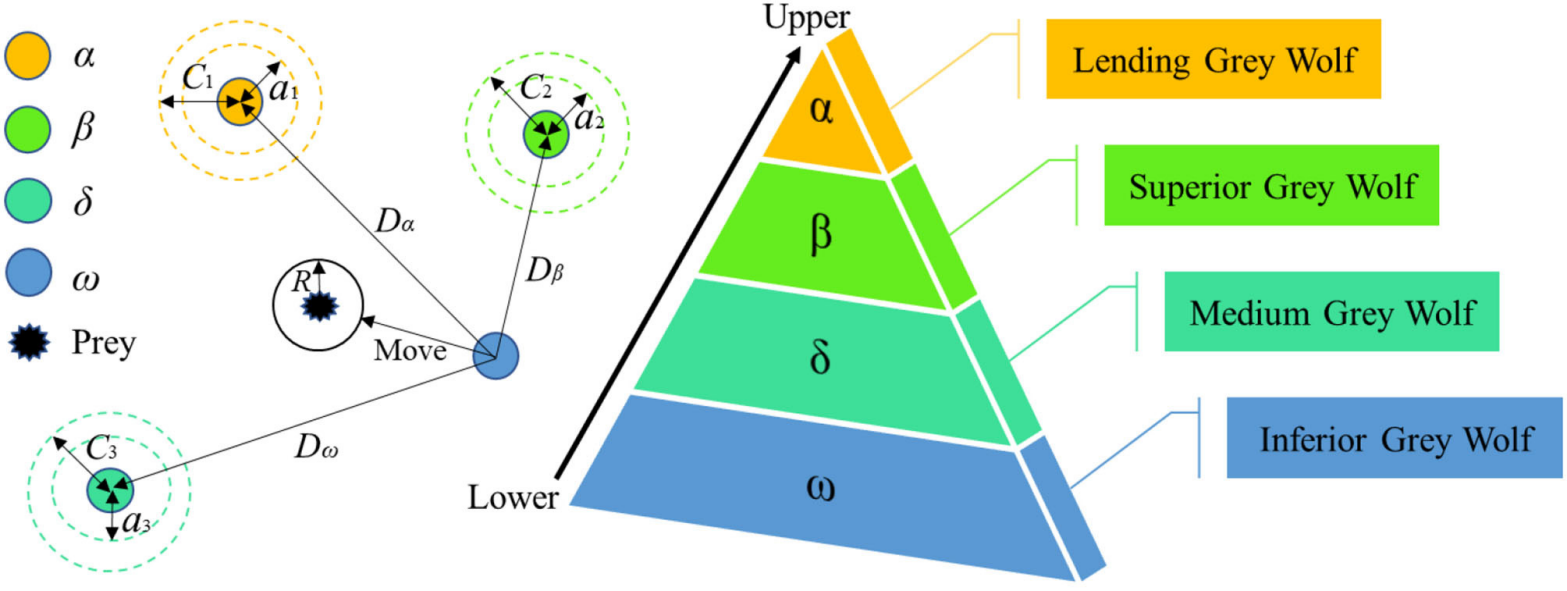
Schematic diagram of GWO.

GWO is a novel swarm intelligence optimization algorithm inspired by the group predation behavior of gray wolves (56), which has been applied in many engineering fields (57–60). There is a strict hierarchy within the gray wolf group, and the entire wolf group is divided into four levels, namely α, β, δ, and ω. As shown in Figure 1, α is the optimal gray wolf, which is the leader of the wolf group and has the right to decide all major issues of the entire wolf group; β is the second-best gray wolf, assisting the leader wolf to make decisions; δ is at the third level, responsible for sentry, reconnaissance and other tasks; ω is the lowest level wolf and is under the command of the first three levels of gray wolves in action. More descriptions of the principle of GWO can be found in the literature (56, 61).

The predation process of gray wolves is divided into three processes: search, surround, and attack. During the hunting process, the individual gray wolf realizes the update of the position according to the following formula to round up the prey.

(4) $$\begin{array}{l} D=|\vec{C}\cdot {\vec{X}}_{p}\left(t\right)-{\vec{X}}_{p}\left(t\right)| \end{array}$$

(5) $$\begin{array}{l} \vec{X}\left(t+1\right)={\vec{X}}_{p}\left(t\right)-\vec{A}\cdot D \end{array}$$

(6) $$\begin{array}{l} \vec{A}=2\vec{a}\cdot {\vec{r}}_{1}-\vec{a} \end{array}$$

(7) $$\begin{array}{l} \vec{C}=2\cdot {\vec{r}}_{2} \end{array}$$

Where, *t* is the current number of iterations; *D* is the distance between the individual gray wolf and its prey; $\vec{X}$ is the position vector of the individual gray wolf; ${\vec{X}}_{p}$ is the position vector of the prey; $\vec{A}$ and $\vec{C}$ are coefficient vectors; $\vec{a}$ is the convergence factor that decreases linearly from 2 to 0 with the number of iterations; ${\vec{r}}_{1}$ and ${\vec{r}}_{2}$ are vectors of random numbers between [0,1].

### 2.3. Butterfly optimization algorithm

BOA is a metaheuristic algorithm proposed based on the foraging and courtship behavior of butterflies (62). Assume that each butterfly releases a certain intensity of scent and the concentration of the released scent is related to its adaptation, while each butterfly senses the scent of other butterflies around it and moves toward those that emit more scent. The intensity of the fragrance produced by butterflies is represented by Equation (8).

(8) $$\begin{array}{l} f_{i}=cT^{\alpha } \end{array}$$

where *f*_*i*_ is the scent intensity perceived by the *i*-th individual butterfly, *c* is the sensory modality of the butterfly, *T* is the stimulus intensity, α is a power exponential parameter that depends on the sensory modality.

When the individual butterfly feels that a certain butterfly emits more fragrance in this area, it will conduct a global search toward the source of the fragrance. This process is represented by Equation (9).

(9) $$\begin{array}{l} x_{i}^{t+1}=x_{i}^{t}+r*\left(g^{*}-x_{i}^{t}\right)*f_{i} \end{array}$$

where $x_{i}^{t}$ is the solution vector of the *i*-th butterfly in the *t*-th iteration; g^*^ represents the optimal solution among all solutions in the current iteration; r is a random number between [0, 1].

When a butterfly cannot perceive a scent higher than its own, it will fly randomly and perform a local search. The local search can be expressed by Equation (10).

(10) $$\begin{array}{l} x_{i}^{t+1}=x_{i}^{t}+r*\left(x_{k}^{t}-x_{j}^{t}\right)*f_{i} \end{array}$$

where $x_{j}^{t}$ and $x_{k}^{t}$ represent the solution vectors of the *j*-th and *k*-th butterflies in the solution space of the *t*-th iteration. If $x_{j}^{t}$ and $x_{k}^{t}$ belong to the same population, and *r* is a random number within (0, 1), it means a local random walk.

### 2.4. Tunicate swarm algorithm

TSA is a metaheuristic algorithm based on the jet propulsion and swarm behavior of tunicates in the ocean during foraging (63). Tunicates use two of their own behaviors to find food sources, jet propulsion and swarm intelligence. A specific description of the principle of the algorithm can be found in the literature (63). For mathematical modeling of jet propulsion behavior, tunicates should satisfy the following three conditions.

Avoid conflicts between search populations. To avoid the conflict between tunicates, TSA calculates the new tunicate position through Equation (11).

(11) $$\begin{array}{l} \vec{X}=\frac{c_{2}+c_{3}-2c_{1}}{\left|V_{\min}+c_{1}\cdot V_{\max}-V_{\min}\right|} \end{array}$$

Where $\vec{X}$ is the new tunic position; *V*_min_ and *V*_max_ are the minimum and maximum initial speed of social interaction, respectively, and are generally set as [1, 4]; *c*_1_, *c*_2_, *c*_3_ are random numbers between [0,1].

Move to the position of the best search individual.

(12) $$\begin{array}{l} \vec{D}=|\vec{F}-r\cdot \vec{P}\left(x\right)| \end{array}$$

where $\vec{D}$ is the distance between the food and the searched individual; $\vec{F}$ is the position of the table food; *x* is the number of iterations; $\vec{P}\left(x\right)$ represents the position of the tunicate individual in the x-th iteration. *r* is a random number between [0,1].

Converge to the optimal position.

(13) $$\begin{array}{l} \vec{P}\left(x^{*}\right)=\{\begin{array}{ll} \vec{F}+\vec{X}\cdot \vec{D}, & r\geq 0.5\\ \vec{F}-\vec{X}\cdot \vec{D}, & r<0.5 \end{array} \end{array}$$

where $\vec{P}\left(x^{*}\right)$ is the updated position.

After modeling the individual jet propulsion behavior, the swarm behavior is modeled. TSA saves the first two optimal solutions and updates the positions of other search individuals according to the position of the best search individual. Group behavior can be defined using Equation (14).

(14) $$\begin{array}{l} \vec{P}\left(x+1\right)=\frac{\vec{P}\left(x\right)+\vec{P}\left(x+1\right)}{2+c_{1}} \end{array}$$

### 2.5. Multi-verse optimizer

MVO is a metaheuristic optimization algorithm, which has been widely used in many fields (64–67). It is based on the principle that matter in the universe is simulated by transferring from a white hole to a black hole through a wormhole, which can be described in the literature (64). Here are some brief introductions about MVO.

Assuming that the universe matrix exists in the search space is:

(15) $$\begin{array}{l} U=\left[\begin{array}{llll} x_{1}^{1} & x_{1}^{2} & \ldots & x_{1}^{d}\\ x_{2}^{1} & x_{2}^{2} & \ldots & x_{2}^{d}\\ \ldots & \ldots & \ldots & \ldots \\ x_{n}^{1} & x_{n}^{2} & \ldots & x_{n}^{d} \end{array}\right] \end{array}$$

Where *d* is the spatial dimension, *n* is the number of universes.

Due to the different inflation rates of each individual universe, the individuals in the universe are able to transfer through white or black holes, chosen randomly using the roulette wheel method, as shown in Equation (16).

(16) $$\begin{array}{l} x_{i}^{j}=\{\begin{array}{ll} x_{k}^{j} & r_{1}<NI\left(U_{i}\right)\\ x_{k}^{j} & r_{1}\geq N_{l}\left(U_{i}\right) \end{array} \end{array}$$

Where $x_{i}^{j}$ is the *j*-th variable of the *i*-th universe, $x_{k}^{j}$ is the *j*-th variable of the *i*-th universe selected by a roulette wheel selection mechanism, *U*_*i*_ shows the *i*-th universe, *NI*(*U*_*i*_) is a normalized inflation rate of the *i*-th universe, *r*_1_ is a random number in [0,1].

The random transport of matter between universes through wormholes to ensure population diversity and the movement of individual universes toward the current optimal universe to increase the inflation rate is given by the following equation.

(17) $$\begin{array}{l} x_{i}^{j}=\{\begin{array}{l} \{\begin{array}{ll} X_{j}+TDR*((ub_{j}-lb_{j})*r_{4}+lb_{j}) & r_{3}<0.5\\ X_{j}-TDR*((ub_{j}-lb_{j})*r_{4}+lb_{j}) & r_{3}\geq 0.5 \end{array}\\ x_{j}^{i}\;r_{2}\geq WEP \end{array} \end{array}$$

where *X*_*j*_ is the *j*-th variable of the optimal position found so far, *ub*_*j*_ and *lb*_*j*_ respectively represent the upper and lower boundaries of the search space where the *j*-th parameter is located, *r*_2_, *r*_3_, and *r*4 are all random numbers between [0, 1]. *WEP* and *TDR* are the wormhole existence probability and the travelling distance rate, respectively, and their expressions are as follows.

(18) $$\begin{array}{l} WEP=WEP_{\min}+l*\left(\frac{WEP_{\max}-WEP_{\min}}{L}\right) \end{array}$$

(19) $$\begin{array}{l} TDR=1-\frac{l^{1/p}}{L^{1/p}} \end{array}$$

Where *WEP*_min_ and *WEP*_max_ are the minimum and maximum values of parameter *WEP, l*, and *L* are the current number of iterations and the maximum number of iterations, *p* is the exploit precision in the algorithm.

### 2.6. Back propagation neural network

The back propagation neural network (BPNN) is a multi-layer network that forwards signals and propagates errors backwards. BP neural network has been studied and applied to solve many problems (68–72), and is one of the most widely used networks at present. It can simulate the information transmission mode of human brain neurons, perform non-linear transformation and regression processing on complex information variables, and obtain operation results with a high fitting degree. The structure of the BP neural network consists of three layers: input layer, hidden layer, and output layer (52). The number of hidden layers is not fixed, it can be one layer or multiple layers.

Suppose the number of nodes in the input layer, hidden layer, and output layer are *l, m, n*. The weight from the input layer to the hidden layer is ω_*ij*_, the weight from the hidden layer to the output layer is $\omega_{jk}^{{\;}^{\prime }}$, the threshold from the input layer to the hidden layer is *q*_*j*_, and the weight from the hidden layer to the output layer is $q_{k}^{{\;}^{\prime }}$. The excitation function *g*(*t*) is taken as a Sigmoid function with the following equation.

(20) $$\begin{array}{l} g\left(t\right)=\frac{1}{1+e^{-t}} \end{array}$$

Then the output of the neuron in each layer is:

(21) $$\begin{array}{l} \{\begin{array}{ll} H_{j}=g\left(\sum_{i=1}^{l}\omega_{ij}x_{i}-q_{j}\right), & j=1,2,\ldots ,m\\ O_{k}=f\left(\sum_{j=1}^{m}\omega_{jk}^{{\;}^{\prime }}H_{j}-q_{k}^{{\;}^{\prime }}\right), & k=1,2,\ldots ,n \end{array} \end{array}$$

where *H*_*j*_ is the output of the *j*th neuron in the hidden layer, *O*_*k*_ is the output of the neural network, and *x*_*i*_ is the input data.

**Figure 2:**
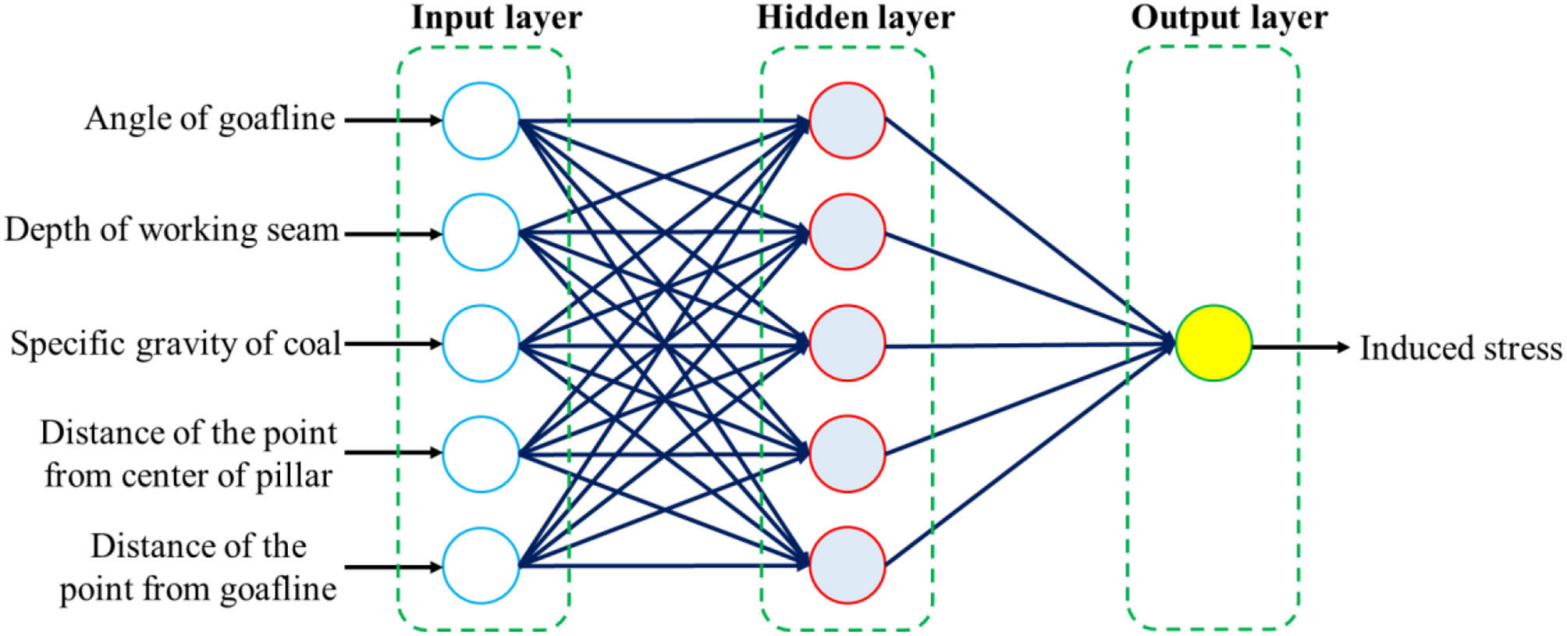
BPNN model framework for estimating induced stress.

Just like the information transmission between neurons in the brain, after the input variables are input in the input layer, a linear combination of the input variables will be obtained according to the initially set weights. When the weights are continuously modified such that the linear combination value exceeds the threshold, the information is passed to the output layer (73). Figure 2 illustrates the BPNN model framework for predicting induced stress at different locations within the pillar in this study based on 5 input variables and 1 output variable.

## 3. Materials

### 3.1. Data description

**Figure 3:**
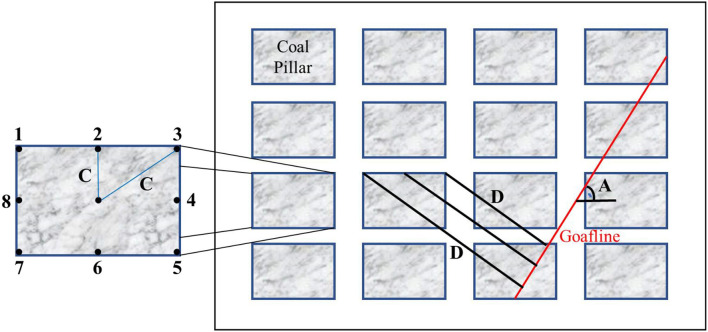
Plan of a sub-panel (2).

The data used in this study refer to reference (2), and the database used is from several coal mines whose geological and geometrical conditions are basically the same and which are excavated by bord and pillar method. The main purpose of this study is to predict the induced stress at different locations within the same pillars. Figure 3 shows the plan of the sub-panel, in which Arabic numerals are used to represent different locations within the same pillars. The parameters affecting the induced stress level in the point are the angle of goafline (A), depth of the working coal seam (H), specific gravity of the coal (G), distance of the point from the center of the pillar (C), and distance of the point from goafline (D). These five parameters have been taken as input variables and the induced stress (S) within the coal pillar point as the output variable in this study.

**Figure 4:**
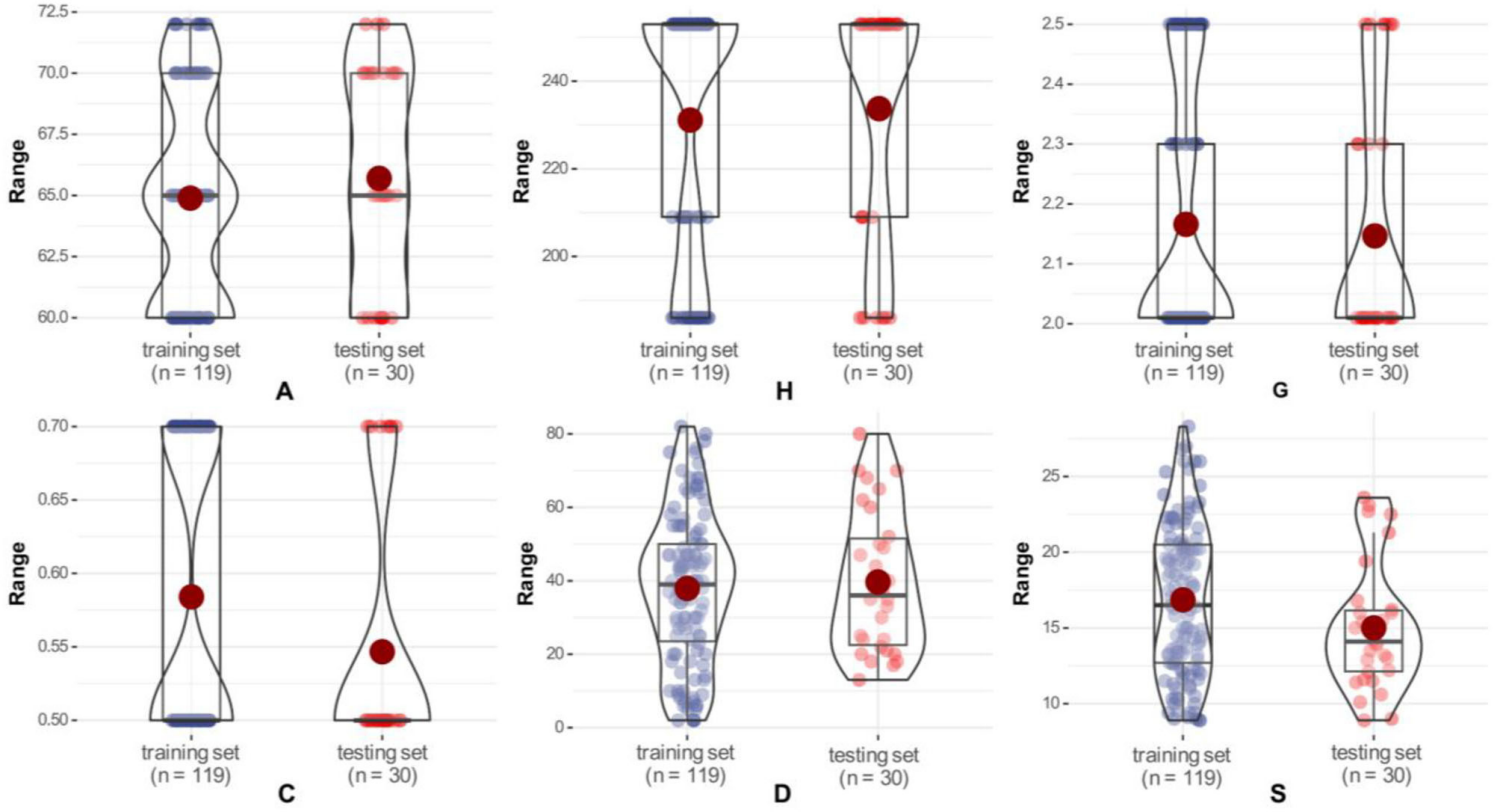
Data distribution of the training set and testing set for the model.

A total of 149 datasets were collected in this study to predict stress magnitudes. Table 1 presents some statistics about the input and output parameters (mean, range, and standard deviation) along with their units and descriptions to better illustrate the used data samples. In addition, in order to train and test the model, the database is randomly divided into a training set and a test set, and their data distribution status is represented by a violin plot, as shown in Figure 4. The violin plot is a combination of a box plot and a kernel density plot, where the black boxes indicate the range from the lower quartile to the upper quartile and the red dots in the middle represent the mean (61). From the figure, it can be seen that the parameter data distributions of the training set and testing set are approximately the same in order to ensure that the results obtained in this study will be reliable.

**Table 1:** Descriptive statistics for input and output parameters.

**Category**	**Symbol**	**Parameter**	**Unit**	**Min**	**Max**	**Mean**	**Std**.
Input	A	Angle of goafline	°	60	72	65.05	4.30
Input	H	Depth of the working coal seam	m	186	253	231.65	29.18
Input	G	Specific gravity	t/m^3^	2.01	2.5	2.16	0.21
Input	C	Distance of the point from the center of the pillar	m	0.5	0.7	0.58	0.10
Input	D	Distance of the point from goafline	m	2	82	38.28	20.19
Output	S	Induced stress	MPa	8.9	28.3	16.48	4.90

**Figure 5:**
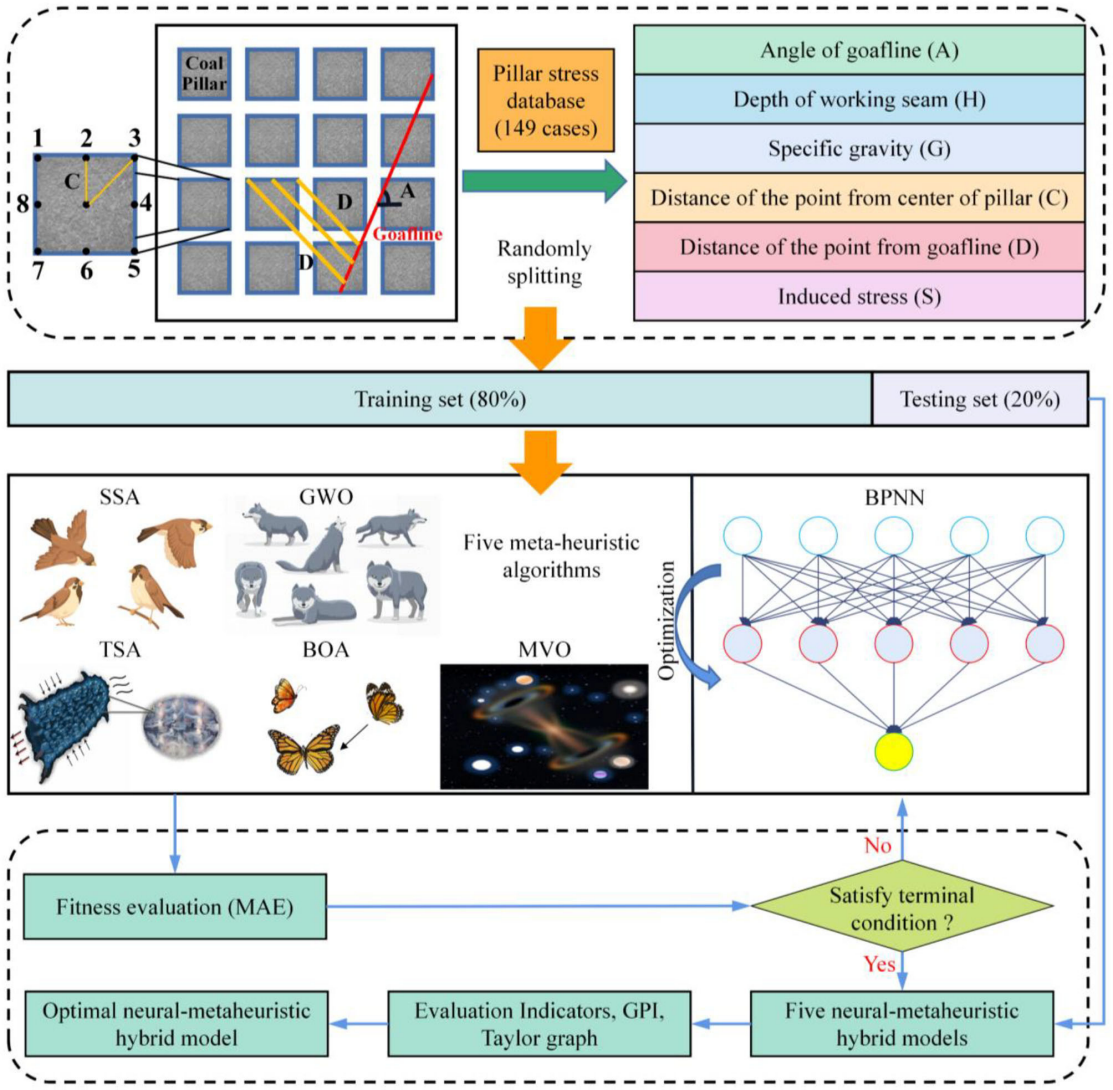
Flowchart of SSA-BP, GWO-BP, BOA-BP, TSA-BP, and MVO-BP prediction models.

The overall analysis and modeling process of the five hybrid models is shown in Figure 5. All data sets were normalized to the range [−1, 1] in order to improve prediction accuracy and avoid redundant computational costs. To build the mixture model effectively, the collected database was randomly divided into 80% training set and 20% test set based on the Pareto principle (74). The weights and thresholds of the BPNN were then optimized using five metaheuristic algorithms, and the mean absolute error (MAE) was used as the fitness value in the optimization process to determine whether to stop, and the optimal weights and thresholds were assigned to the network predictions. Finally, the predictive performance of the developed neural-metaheuristic hybrid model was evaluated for the training and test sets using five evaluation indicators (R^2^, RMSE, VAF, MAE, and MAPE).

### 3.2. Model validation and evaluation

#### 3.2.1. Evaluation indicators

Validation and evaluation of model performance play an important role in the model building process. Therefore, in order to effectively evaluate the quality of five hybrid prediction models, namely SSA-BPNN, GWO-BPNN, TAS-BPNN, BOA-BPNN, and MVO-BPNN, this study adopts coefficient of determination (R^2^), root mean squared error (RMSE), variance accounted for (VAF), mean absolute error (MAE), and mean absolute percentage error (MAPE) as the evaluation indicators of the models. Generally, R^2^ and VAF values equal to 100, and RMSE, MAE, and MAPE values equal to 0 indicate the best predictive performance of a model. The interpretation of these indicators is given in the literature (75–89), and their expressions are shown in Equations (22–26).

(22) $$\begin{array}{l} {\text{R}}^{2}=1-\frac{\overset{n}{\underset{i}{\sum }}{\left(S_{i}-{\hat{S}}_{i}\right)}^{2}}{\overset{n}{\underset{i}{\sum }}{\left(S_{i}-{\bar{S}}_{i}\right)}^{2}} \end{array}$$

(23) $$\begin{array}{l} \text{RMSE}=\sqrt{\frac{1}{n}\overset{n}{\underset{i=1}{\sum }}{\left(S_{i}-{\hat{S}}_{i}\right)}^{2}} \end{array}$$

(24) $$\begin{array}{l} \text{VAF}=\left[1-\frac{var\left(S_{i}\text{-}{\hat{S}}_{i}\right)}{var\left(S_{i}\right)}\right]\times 100 \end{array}$$

(25) $$\begin{array}{l} \text{MAE}=\frac{1}{n}\overset{n}{\underset{i=1}{\sum }}|S_{i}-{\hat{S}}_{i}| \end{array}$$

(26) $$\begin{array}{l} \text{MAPE}=\frac{1}{n}\overset{n}{\underset{i=1}{\sum }}\frac{\left|S_{i}-{\hat{S}}_{i}\right|}{S_{i}}\times 100 \end{array}$$

where *n* is the number of input-output data pairs, *S* is the actual column stress, Ŝ is the predicted column stress, and $\bar{S}$ is the average value of the actual column stress.

#### 3.2.2. Global performance indicator

To avoid complex and tedious integration of scoring rankings, this study uses the global performance indicator (GPI) for the comprehensive evaluation of the model (75, 90). Before calculating the GPI, each evaluation indicator needs to be normalized in the interval [0, 1], after calculating according to the following equation.

(27) $$\begin{array}{l} \text{GP}{\text{I}}_{i}=\overset{5}{\underset{j=1}{\sum }}\beta_{j}\left({\tilde{y}}_{j}-y_{ij}\right) \end{array}$$

where β_*j*_ is equal to 1 for RMSE, MAE, and MAPE, and −1 for R^2^ and VAF. ỹ_*j*_ is the median of the scaled values of indicator *j* and *y*_*ij*_ is the scaled value of indicator *j* of model *i*. The less the RMSE, MAE, and MAPE are than the median value, and the higher the R^2^ and VAF are above the median value, the higher the GPI value is. It can be seen that the better the performance of the model, the higher its GPI value.

## 4. Results and discussion

### 4.1. Determination of the optimal population size for the model

**Figure 6:**
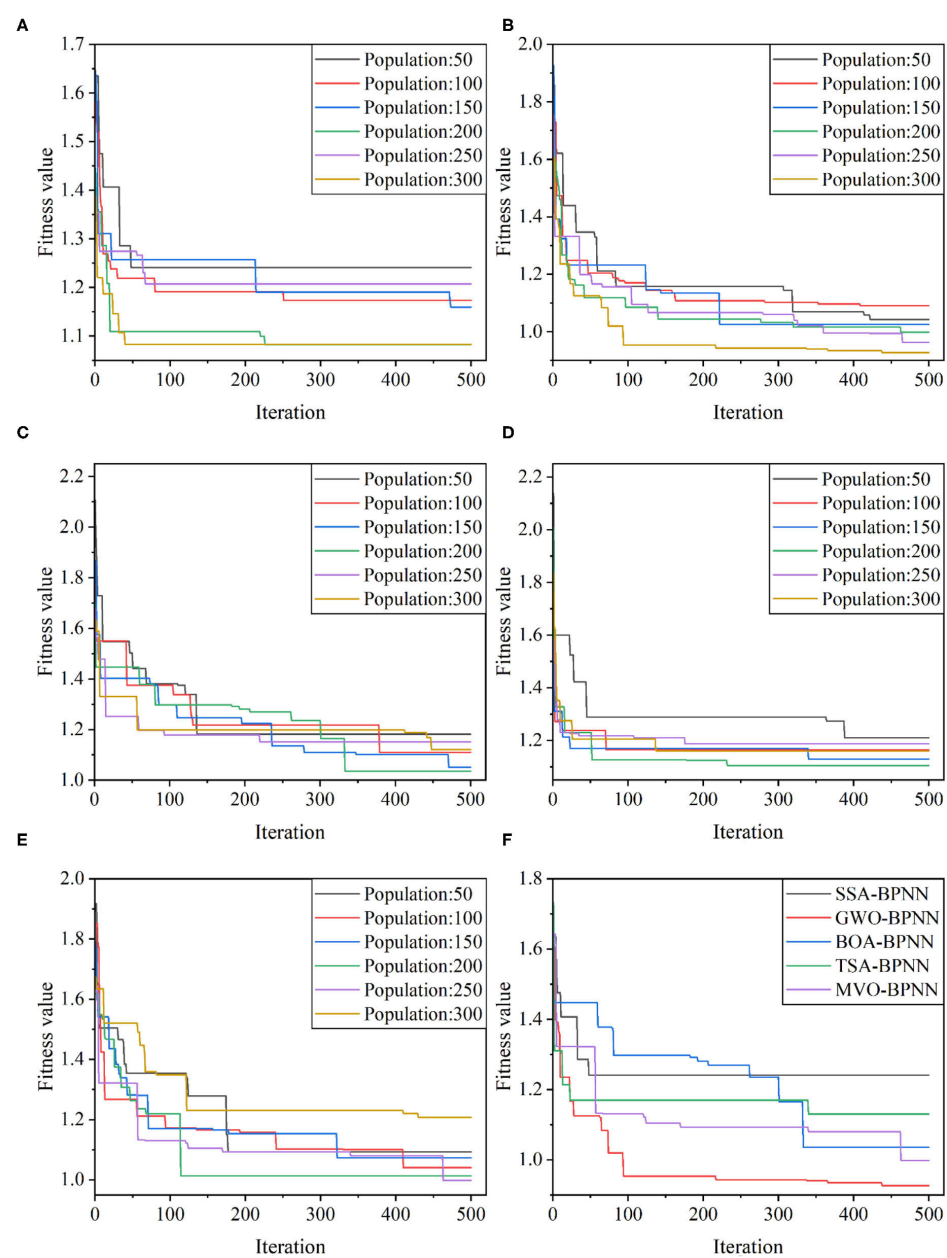
Changes in the fitness values of hybrid models: **(A)** SSA-BP; **(B)** GWO-BP; **(C)** BOA-BP; **(D)** TSA-BP; **(E)** MVO-BP; **(F)** the fitness values of five different optimal population size models.

To determine the optimal weights and thresholds to obtain the best column stress prediction performance, five meta-heuristic algorithms (SSA, GWO, BOA, TSA, and MVO) were used to tune the weights and thresholds in the BPNN model, and the mean absolute error (MAE) was used as the fitness. In this study, six population sizes of 50, 100, 150, 200, 250, and 300 were designed for each of the five hybrid models. the maximum number of iterations was set to 500 to select the best optimal parameters. Figures 6A–E shows the fitness curves of the five hybrid models with different population sizes, and it can be seen that the optimal process is different for different population sizes, and the MAE value decreases with the increase of the number of iterations. The lowest fitness values of SSA-BPNN, GWO-BPNN, BOA-BPNN, TSA-BPNN, and MVO-BPNN were 1.0827, 0.92683, 1.03569, 1.10515, and 0.99775, corresponding to the mixed models with population sizes of 200, 300, 200, 200, and 250, in that order. It indicates that these hybrid models perform best in the training set. Of course, the fitness curve alone is not sufficient to determine the optimal population size. Therefore, to determine the optimal population size, we evaluated the model performance with five indicators and calculated the GPI values of each model based on the evaluation indicators, and the results are shown in Table 2. Better models have higher GPI values, so the optimal population size of SSA-BPNN is 50 (Training set: R^2^ = 0.9984, RMSE = 0.2009, VAF = 99.84, MAE = 0.1262, and MAPE = 0.8477; Test set: R^2^ = 0.9980, RMSE = 0.1846, VAF = 99.80, MAE = 0.1400, and MAPE = 1.0736), the optimal population size of GWO-BPNN is 300 (training set: R^2 =^ 0.9991, RMSE = 0.1535, VAF = 99.91, MAE = 0.0884, MAPE = 0.6107; test set: R^2 =^ 0.9983, RMSE = 0.1783, VAF = 99.83, MAE = 0.1230, and MAPE = 0.9253), the optimal population size of BOA-BPNN is 200 (training set: R^2^ = 0.9988, RMSE = 0.1750, VAF = 99.88, MAE = 0.0934, and MAPE = 0.6607; test set: R^2^ = 0.9973, RMSE = 0.2207, VAF = 99.71, MAE = 0.1543, and MAPE = 1.1900), and the optimal population size of TSA-BPNN is 150 (Training set: R^2^ = 0.9989, RMSE = 0.1667, VAF = 99.89, MAE = 0.1041, and MAPE = 0.7292; Test set: R^2^ = 0.9975, RMSE = 0.2234, VAF = 99.75, MAE = 0.1642, and MAPE = 1.2421), the optimal population size of MVO-BPNN is 250 (training set: R^2^ = 0.9989, RMSE = 0.1692, VAF = 99.88, MAE = 0.1076, and MAPE = 0.7078; test set: R^2^ = 0.9968, RMSE = 0.2498, VAF = 99.67, MAE = 0.1754, and MAPE = 1.2480).

**Table 2:** GPI values for different population sizes of the hybrid models.

		**SSA-BPNN**	**GWO-BPNN**	**BOA-BPNN**	**TSA-BPNN**	**MVO-BPNN**

GPI	50	3.029	−5.639	−1.966	−3.635	2.322
	100	−0.618	−0.012	1.018	−1.155	0.257
	150	−3.346	−3.845	1.099	4.861	−0.535
	200	−3.929	1.454	3.686	0.331	−3.231
	250	2.218	1.300	0.959	1.956	5.264
	300	0.429	3.289	−5.667	1.444	−2.103

### 4.2. Comparative analysis of hybrid models

**Figure 7:**
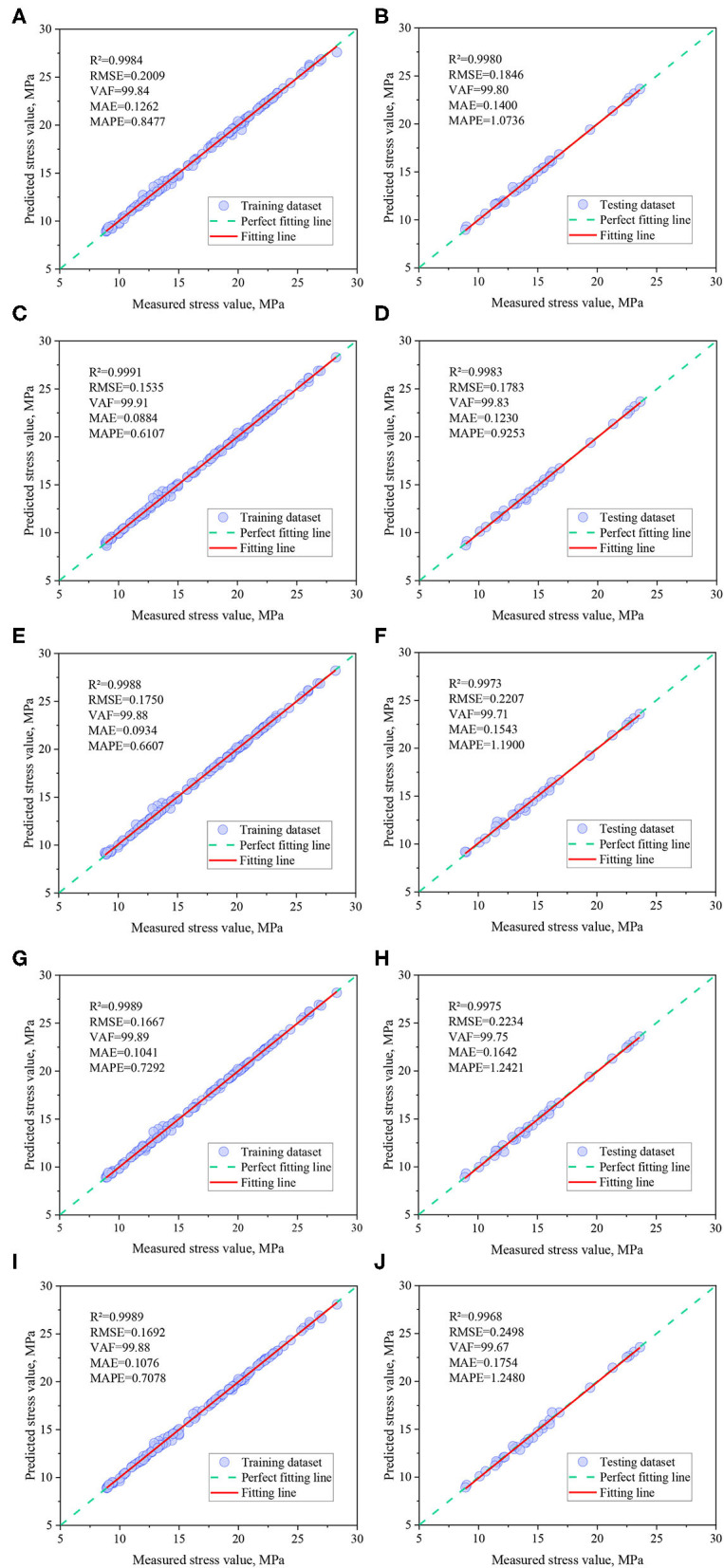
Correlation analysis between measured and predicted induced stress. **(A)** SSA-BP (Training). **(B)** SSA-BP (Testing). **(C)** GWO-BP (Training). **(D)** GWO-BP (Testing). **(E)** BOA-BP (Training). **(F)** BOA-BP (Testing). **(G)** TSA-BP (Training). **(H)** TSA-BP (Testing). **(I)** MVO-BP (Training). **(J)** MVO-BP (Testing).

The correlations between predictions and measured induced stresses for the five optimal mixed model training and test datasets are shown in Figure 7. It can be seen from the figure that the training and testing sample points of these hybrid models are basically distributed near the perfect fitting line (“measured stress value = predicted stress value”), and the R^2^ values are above 0.99, indicating that the five optimization techniques based on BP neural network proposed in this paper can achieve high training and testing outcome results.

**Figure 8:**
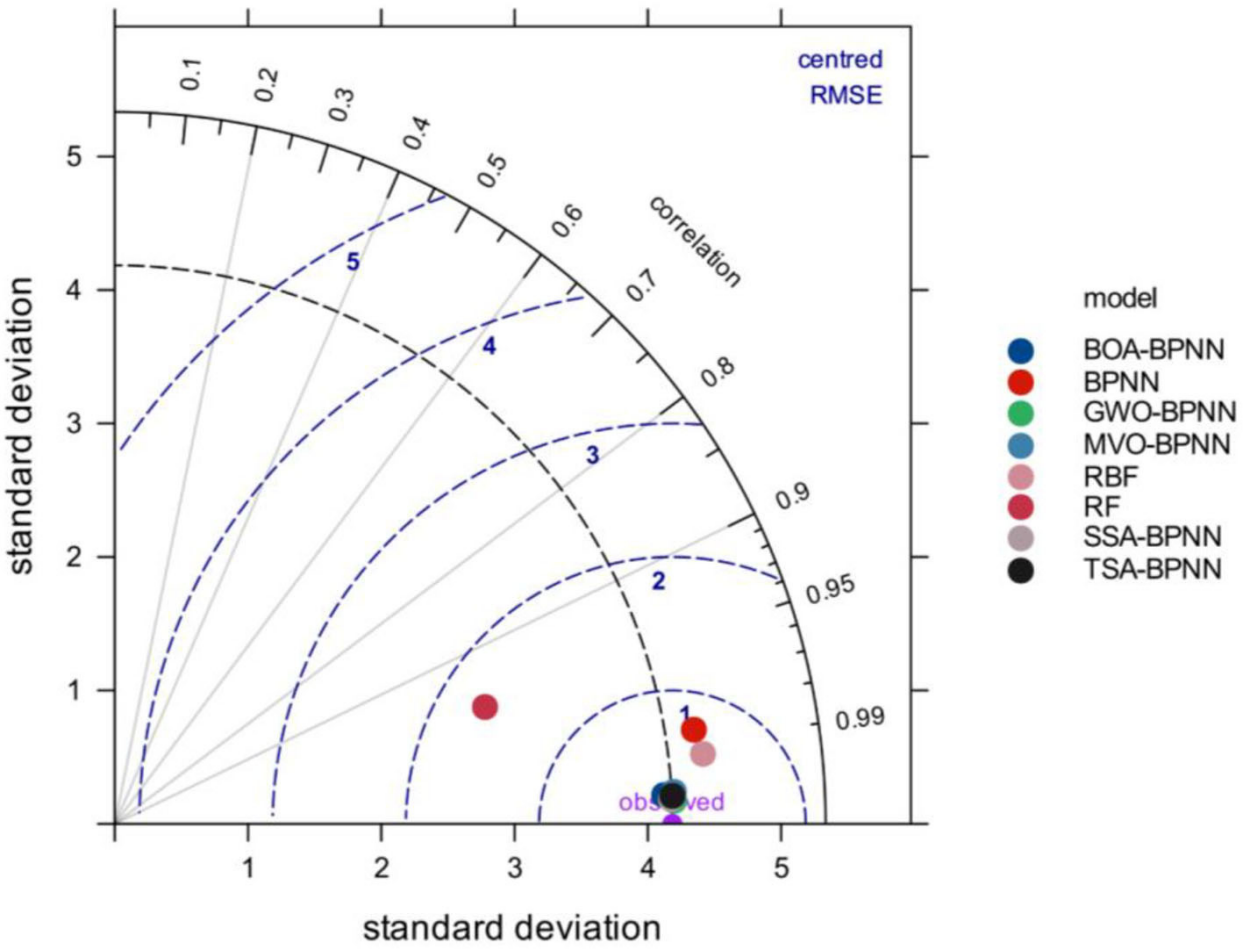
Model performance comparison in Taylor graph.

In order to compare and evaluate the prediction performance of the hybrid models, based on the previous discussion, three classical machine learning models, BPNN, random forest (RF) and radial basis function network (RBF), were also introduced in this study for comparison, and the GPI values of each model were calculated, and the detailed data are listed in Table 3. from the table, it can be seen that the GWO-BPNN model has the best prediction performance (on the training set, R^2^ = 0.9991, RMSE = 0.1535, VAF = 99.91, MAE = 0.0884, and MAPE = 0.6107; on the test set, R^2^ = 0.9983, RMSE = 0.1783, VAF = 99.83, MAE = 0.1230, and MAPE = 0.9253). Figure 6F gives an iterative graph of the objective optimization of the five hybrid models, from which it can be seen that the GWO-BPNN hybrid model shows the best results on the training set. In addition, to further demonstrate the superiority of the hybrid model, the Taylor graph is utilized to more visually show the performance of the model on the test set (as shown in Figure 8). The Taylor graph shows the correlation coefficient, RMSE, and standard deviation between the actual and predicted values of the model (65, 75, 91–93). The Taylor graph shows that the hybrid model significantly outperforms the un-optimized classical model. In conclusion, while all hybrid models performed well in predicting induced stress with high accuracy, the GWO-BPNN showed the best predictive performance overall. It is therefore recommended to apply GWO-BPNN to predict the induced stress at different locations within the pillar.

**Table 3:** Ranking of different models according to GPI.

	**SSA-BPNN**	**GWO-BPNN**	**BOA-BPNN**	**TSA-BPNN**	**MVO-BPNN**	**BPNN**	**RF**	**RBF**
GPI	0.060	0.191	0.072	0.056	0.017	−2.210	−9.809	−2.469

It is important to note that the data used in this article are from a study conducted with Monjezi et al. (2). They developed an ANN model to predict induced stress at different locations in pillar and pillar recovery. They report that in the testing phase, the optimal ANN model has an R^2^ value of 0.988, an RMSE value of 0.056233, an MAE value of 0.309281, and a MAPE value of 2.337007. Compared with this study, the proposed GWO-BPNN model has an R^2^ value of 0.9983, RMSE value of 0.1783, MAE value of 0.1230, and MAPE value of 0.9253, which is better than the ANN model developed by Monjezi et al. (2).

## 5. Conclusions

Estimation of pillar stress plays an important role in determining pillar size, room width and roof conditions. Thus, in order to predict the induced stress at different positions within the coal pillar in room-and-pillar mining method, five hybrid models based on BPNN and metaheuristic algorithms were constructed in this study. The five hybrid models (SSA-BPNN, GWO-BPNN, BOA-BPNN, TSA-BPNN, and MVO-BPNN) were trained and tested using the established database. The angle of goafline (A), Depth of the working coal seam (H), specific gravity (G), distance of the point from the center of the pillar (C), and distance of the point from goafline (D) were taken as input variables, whereas the induced stress (S) were taken as output variable. Model prediction performance was evaluated using five evaluation indicators, GPI, and Taylor diagrams. The experimental results show that the prediction performance of the five hybrid models is significantly better than that of the un-optimized models, and were better than the prediction results of the earlier developed ANN model developed. Among them, the GWO-BPNN model performed the best in both the training phase and the testing phase (Training set: R^2^ = 0.9991, RMSE = 0.1535, VAF = 99.91, MAE = 0.0884, MAPE = 0.6107; Test set: R^2^ = 0.9983, RMSE = 0.1783, VAF = 99.83, MAE = 0.1230, MAPE = 0.9253).

It is worth noting that the prediction model developed in this study can only be used to predict the induced stress in the mine pillar, but the algorithms used can be applied to other engineering practices. In addition, the limitation of using the mixed model to predict induced stress in this research is that the data set is small, and the modeling process only involves 149 cases. In the future, a database with more samples and features needs to be established to ensure the performance and stability of the prediction model.

## Data availability statement

The raw data supporting the conclusions of this article will be made available by the authors, without undue reservation.

## Author contributions

JZ: validation, resources, formal analysis, visualization, software, writing—review and editing, supervision, and funding acquisition. YC: conceptualization, methodology, validation, software, investigation, visualization, and writing—original draft. HC: validation, formal analysis, writing—review and editing, and supervision. MK: formal analysis, validation, visualization, and writing—review and editing. MM: data curation, visualization, and writing and editing. KP: formal analysis, validation, investigation, and writing—review and editing. All authors contributed to the article and approved the submitted version.
